# A New Origin for Entropy: Jauch’s Conservation Law and the Geometry of Thermodynamics

**DOI:** 10.1007/s10701-026-00946-6

**Published:** 2026-09-02

**Authors:** Bryan W. Roberts

**Affiliations:** Philosophy, Logic & Scientific Method, London School of Economics & Political Science, London, England

**Keywords:** Foundations of thermodynamics, Entropy and temperature, Thermodynamic irreversibility, Gauge theory, Ehresmann connections, Jauch’s conservation law

## Abstract

A central question for the foundations of thermodynamics is which conceptual structures underpin the existence of entropy and temperature. Jauch claimed that entropy and temperature could be derived from a novel conservation law. This paper reconstructs the physics and mathematics of Jauch’s claim and finds that his original proof is not valid. Remarkably, his theorem is still true. We provide an alternative proof using geometric ideas from modern gauge theory, revealing a deep geometric structure in thermodynamics. This result also helps to settle an old debate by showing that entropy and temperature can be defined in a new, conceptually clear way without appeal to an irreversible assumption like the second law.

## Introduction

There is an old debate about whether thermodynamic entropy and temperature exist because of an assumption of irreversibility like the second law, or because of facts about adiabatic accessibility [8, 11, 22], or because of some other reason entirely. This is a pressing question for the philosophical foundations of thermodynamics because of the central role that entropy and temperature play in both its conceptual structure and its application across the sciences. Its answer also has an important theoretical component, in part due to the difficulty of defining thermodynamic entropy and temperature using operational measurements.^1^ Albert [1] follows most textbooks in adopting the second law approach, while other philosophers defend the generality and precision of the other approaches [cf. 21, 35]. The debate also has implications for whether entropy is intrinsically linked to a thermodynamic arrow of time.^2^ However, few have considered the implications of an alternative approach published in this journal by Josef-Maria Jauch [17], who claimed that thermodynamic entropy and temperature can be derived from a conservation law.^3^  

Jauch’s conservation law states roughly that if an adiabatic process restores all the work configuration variables to their initial values, then that process cannot perform work. Jauch gave this principle some physical motivation and a precise mathematical expression, pointing out that it does not contain any irreversible ingredient like the second law. He also expressed the hope that it would provide a novel foundation for thermodynamics that frees us once and for all from the “wrong impression that the existence of entropy and temperature are characteristic consequences of irreversibility” or the second law [17, p.328]. Jauch’s new foundation may not be widely known, but it has still been mentioned and interpreted by many authors.^4^ A development of his idea appears in Part I of a treatise published just after his untimely death [18]. No second part was ever published.

My purpose in this paper is to reconstruct the physics and mathematics of Jauch’s proposal, in order to correct and complete it. I first develop the physical and mathematical basis for Jauch’s conservation law, arguing that it is indeed physically plausible and, unlike the second law, contains no irreversible ingredient. Unfortunately, a correction is also needed: Jauch’s derivation of entropy and temperature from this conservation law is not valid, and his argument strategy cannot be straightforwardly repaired. Nevertheless, I will show that Jauch’s theorem is actually true. The alternative proof that I will provide begins by showing how standard thermodynamics can be studied using tools from Yang-Mills gauge theory,^5^ and then uses those tools to reformulate Jauch’s theorem as the requirement of a flat connection on a fibre bundle describing thermodynamics. This reformulation renders the proof a straightforward consequence of the Frobenius theorem, using a different argument from Carathéodory’s theorem. The result is a promising new foundation for thermodynamics, and in particular for the origin of thermodynamic entropy and temperature, which helps to clarify its geometric structure along the way.

I begin in Section 2 by explaining the physics and mathematics of Jauch’s conservation law, and then turn to his derivation in Section 3 to show where it falters. In Section 4 I introduce the geometric structure needed to reformulate Jauch’s conservation law, and then use it to provide a corrected proof. Section 5 then briefly discusses the relationship between this theorem and the work by Tatiana Afanassjewa [11], before concluding in Section 6.

## A Conservation Law

Jauch’s aim is to derive thermodynamic entropy and temperature in a way that avoids the difficulties of the standard ‘Carnot cycle’ derivation. His work is thus in the tradition of Carathéodory, who had a similar aim.^6^ However, instead of beginning with Carathéodory’s principle, Jauch begins with a conservation law. This conservation law is Jauch’s main idea, and indeed is retained entirely in the corrected proof that I will present below. I thus begin by explaining it in some detail: by first giving it some physical motivation, and then providing a formal mathematical statement.

### Physical Motivation

**Fig. 1:**
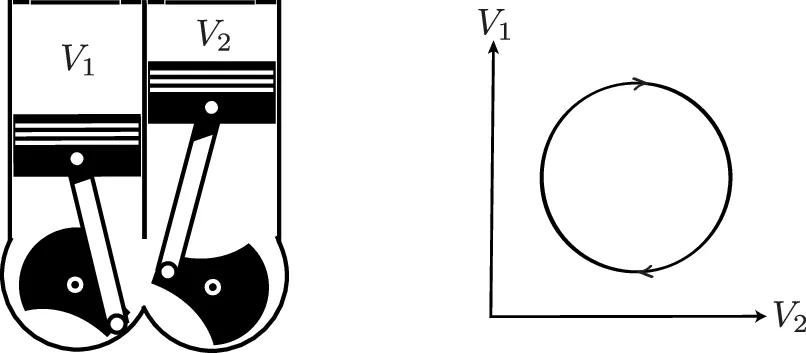
A cyclic engine with two volume variables

Imagine an engine with two cylinders, and thus with work described by two volume variables $V_1,V_2$. As the engine cycles through its various volume states, it traces out a closed loop in the space of work variables called a *work cycle* (Fig. 1).

**Fig. 2:**
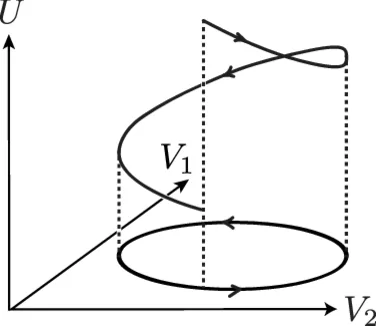
During an engine’s cycle, energy does not return to its initial value even when volume does

During such a cycle, the engine’s energy *U* does not necessarily return to its initial value. Instead, it may exchange energy with its environment, either by performing work or by exchanging heat. So, when the energy of the engine is included in the configuration space, its trajectory forms a downward spiral. The fact that the engine performs a work cycle is reflected in the fact that this spiral projects down onto a closed curve in the plane of work variables (Fig. 2).

This downward spiral can be motivated by Kelvin’s Principle, which I will state as follows (cf. [7], p.89):

*a cyclic process cannot absorb heat and convert it entirely into work.*

Applied to our engine, this means that the process^7^ of absorbing heat to perform work must be limited in a way that leads our engine to lose heat to its environment. The result is an overall decrease in energy, which means the curve in Fig. 2 can spiral down, but it cannot spiral up. Thus, Kelvin’s Principle famously captures an ‘irreversible’ aspect of realistic engines, that they can traverse this curve in one direction but not the other.

Jauch’s conservation law concerns the special case of a cyclic process with no heat exchange whatsoever, which is to say the process is *adiabatic*. In conservative systems, no cyclic process can produce an overall change in energy. But, if a process is adiabatic, then it is only through a change in overall energy that work can be performed. In that case, the spirit of Kelvin’s Principle would be to say: without heat exchange, a cyclic process can do no work. Work energy must come from somewhere, and so if it does not come from heat or a change in energy, then it cannot be produced. This is what I will call *Jauch’s conservation law:*

*a cyclic process without heat exchange cannot perform work.*

This assumption is implicit throughout Carnot’s famous *Reflexions on the Motive Power of Fire*: if a heat-engine receives no heat, then it cannot produce motive power or work.^8^ A consequence of it is that, if a cyclic engine is adiabatic, then it cannot result in any change in total energy. In terms of our diagram, this says that if an adiabatic curve produces a closed loop in the space of work variables, then the curve must be closed. Thus, although Jauch’s conservation law may be viewed as inspired by Kelvin’s Principle, it does not introduce any irreversible ingredient into thermodynamics. For this reason, Jauch refers to it as “the static form of what is usually called the second principle of thermodynamics”, noting that it “refers only to adiabatic processes, hence it is weaker than the second [Kelvin’s] principle” [18, pp.122-3].

This is not to say that Jauch’s conservation law only applies to adiabatic processes. The statement is conditional: *if* a cyclic process is adiabatic, *then* it cannot perform work. That still allows processes represented by curves that are non-adiabatic, and the revision of Jauch’s proof proposed here will explicitly include them. The systems outside the scope of Jauch’s conservation law are rather those that are *non-conservative*, as Jauch himself remarks:

“There are actually systems for which the condition... is not satisfied. They are systems with a certain kind of internal friction such as hysteresis and electrical resistance. In these cases, ordinary thermodynamics is not applicable because reversible adiabatic paths do not exist.” [17, p.330]

We will return to this question of scope at the end of the next subsection. But first, let us make these ideas a little more precise.

### Formal Statement of the Conservation Law

To state Jauch’s conservation law mathematically, let *M* be a smooth connected manifold of dimension *n* with local coordinates $(U,V_1,\dots ,V_{n-1})$, where *U* represents energy and the $V_i$ represent work configuration variables. The full phase space of thermodynamics is usually taken to be a larger *(2n+1)*-dimensional manifold with both intensive and extensive variables; however, it is generally equipped with *n* equations of state which, together with the first law, constrain physical behaviour to an *n*-dimensional manifold like this one.

For some set of smooth real-valued functions $P_1,\dots ,P_{n-1}$ on *M*, let *work* be defined^9^ by $\omega :=-\sum _{i=1}^{n-1}P_idV_i$ and let *heat* be $\xi = dU + \omega$. A curve $\gamma :[0,1]\rightarrow M$ will be called an *adiabat* if there is no heat along it: $\xi (\bar{\gamma })=0$, where $\bar{\gamma }$ is the tangent vector field to *γ *. A curve will be called a *work cycle* just in case it is closed in the work variables, in that for *i=1,… ,n-1*

1 $$\begin{aligned} V_i(\gamma (0)) = V_i(\gamma (1)). \end{aligned}$$

A work cycle is not necessarily a closed curve in *M*, which would require in addition that $U(\gamma (0))=U(\gamma (1))$. Now Jauch’s conservation law, that a cyclic process without heat exchange cannot perform work, can be stated in local terms:

($C_1$): in a neighbourhood *B* of every point, if *γ * is an adiabat and a work cycle contained in *B*, then $\int _{\gamma }\omega =0$.

To unpack this a little: suppose some work cycle is described by an adiabat *γ *. Then, since there is no heat exchange, the first law requires that any work *ω * along the curve is accompanied by a change in energy. Now, if the system in question is ‘conservative’ in the sense that there is nowhere else for energy to enter or exit the system, then such a change in energy (and thus such a change in work) is impossible. This is exactly what Jauch’s conservation law says: on an adiabatic work cycle, no work production is possible. It is in this sense that, as we remarked in the previous subsection, Jauch’s conservation law restricts attention to systems that are conservative.

Jauch’s own terminology is slightly awkward here, but is worth recording: Jauch refers to what I have called a work cycle as a closed curve, despite the fact that it is not closed in *M* in the ordinary topological sense. He later adjusts the terminology to refer to a work cycle as ‘closed’ (with scarequotes) [18, p.122]. I will continue to use the term ‘work cycle’ in what follows to avoid confusion, but Jauch’s terminology is needed to understand his own statement of his conservation law in the paper, where he also writes *δ A* (‘Arbeit’) for the work one-form that I have called *ω *:

“The fundamental hypothesis that characterizes conservative thermal systems is that for every closed [in the sense of being a work cycle] adiabatic *c*, $\int _c \delta A = 0$” [17, p.329].

## Jauch’s Proof

Jauch’s proof is an attempt to derive entropy and temperature, so let me briefly recall why this is a central problem in the foundations of thermodynamics. When work has the form *PdV*, a non-vanishing heat one-form can always be locally expressed as *TdS*: this follows immediately from the existence and uniqueness theorems for ordinary differential equations.^10^ However, this is not generally true when work includes more than one volume variable like $\omega = -P_1dV_1 - P_2 dV_2$. Then there need not exist any smooth functions *T* and *S* such that *ξ =TdS*. So, a further postulate is needed to describe the general circumstances under which this is the case. Jauch, Carathéodory, and many others are concerned with the question of which physical postulate is appropriate for that purpose, and which is a necessary condition for the conclusion that entropy and temperature exist. Following that literature, I will refer to such functions *T* and *S* as temperature and entropy in this paper, although further properties may be desirable to recover other familiar properties of these quantities.

Jauch’s claim is that his conservation law, which he calls *hypothesis (1)*, is the appropriate physical postulate. I have adopted a more standard notation than him, so I will briefly summarise the differences before quoting him. Jauch denotes the configuration space manifold by $\mathcal {X}$ instead of *M*, and takes its dimension to be *n+1* instead of *n*. His local coordinates are $(x_0,x_1,\dots ,x_{n})$ instead of $(U,V_1,\dots ,V_{n-1})$, with energy denoted by $x_0$ instead of our *U*. Finally, Jauch writes heat as *δ Q* instead of *ξ * and work as *δ A* instead of *ω *. Jauch then claims:

“**Theorem.** Under hypothesis (1), the differential form $\delta Q = dx_0 + \delta A$ admits an integrating factor *T* in a suitable neighbourhood of any point in $\mathcal {X}$ so that *δ Q = TdS* where *S* is some function” [17, p.329].

In our notation, this says: *given *$(C_1)$*, every point admits a neighbourhood in which *$\xi = dU + \omega$* is equal to TdS for some smooth functions T and S.*

Jauch’s argument proceeds as follows. Fixing a point *a∈ M*, Jauch defines a function $\mathcal {U}$ by,

2 $$\begin{aligned} \mathcal {U}(x) := \int _a^x \omega + \text {constant} \end{aligned}$$

where for each *x* the integral is taken along any adiabat *γ * from *a* to *x* [17, Equation 2]. If there is more than one such adiabat, Jauch points out that they will nevertheless produce the same value for $\mathcal {U}(x)$. This is where Jauch makes use of his conservation law $(C_1)$: since an adiabatic work cycle *c* satisfies $\int _c\omega =0$, it follows that for the adiabat *γ * in (2), “the value of the integral is independent of path (as long as it is an adiabat)” [17, p.330]. This is the main step in the proof. The function $\mathcal {U}$ is then used as the basis for a coordinate transformation on the neighbourhood, which is then in turn used to construct entropy and temperature functions.

Unfortunately, the main step is not valid. Although Jauch addresses the possible non-uniqueness of adiabatic paths in defining $\mathcal {U}$, he does not address their possible non-existence: there might be points *x* and *a* that are not adiabatically connected. Without this assurance, the function is not defined on the entire neighbourhood, as would be required to use it as Jauch does to define a new coordinate in a coordinate transformation.

The problem can be made stark by asking, what is the domain of $\mathcal {U}$? It is not clear from Jauch’s writing. However, three lines above the definition of $\mathcal {U}(x)$, he defines *x* to be an arbitrary point in the neighbourhood. This is problematic: it implicitly assumes each point *x* is connected to *a* by an adiabat, whereas Carathéodory’s principle says that there are points in every neighbourhood that are *not* adiabatically connected to *a*. The problem is that Carathéodory’s principle is known to be equivalent^11^ to Jauch’s conclusion, that there exist smooth functions *T*, *S* in a local neighbourhood of every point such that *ξ = TdS*. So, Jauch’s argument appears to introduce an implicit assumption that is incompatible with his conclusion.

Even if Jauch were to accept that $\mathcal {U}$ is not defined on the entire neighbourhood, it then cannot be straightforwardly used to define a coordinate function on that neighbourhood, and so the rest of the argument falls apart. As a potential fix, one might hope to define a foliation of the neighbourhood into adiabatically connected hypersurfaces $r\mapsto \Sigma _r$. The hope would be to define a one-parameter *set* of functions $\mathcal {U}_r(x) = \int _a^x\omega$, each defined for all $x\in \Sigma _r$, and then to use this one-parameter set to define $\mathcal {U}$. However, Jauch has given no indication of how such a foliation could be constructed, or why each surface is guaranteed to be adiabatically connected. Such statements rather appear to require the very integrability condition that Jauch is trying to prove.

In my view, there is no straightforward repair to this proof strategy. Remarkably, Jauch’s theorem does still turn out to be true. But, the proof that I will give of this fact makes use of an entirely different strategy, which is the subject of the next section.

## Corrected Proof

This section proceeds in three stages. First, I reformulate thermodynamics on a fibre bundle, in order to generalise Jauch’s separation of volume and energy variables in thermodynamics. Second, in that language, I give two equivalent reformulations of Jauch’s conservation law that help to clarify its mathematical significance. Finally, I show that these reformulations provide an elegant proof of Jauch’s theorem that avoids the problems with his original argument.

### Fibre Bundle Framework

The configuration space of our engine in Fig. 2 involves two manifolds: the 2-dimensional manifold in which a loop is traced in volume space, and the 3-dimensional manifold that includes energy, and in which the complete trajectory of the engine is a spiral. Every system in thermodynamics has these two general components: an *(n-1)*-dimensional manifold *N*, whose points are the observable configurations characterising work, and an *n*-dimensional manifold *M*, with an added dimension describing total energy (internal energy). These two manifolds are related by a smooth projection,

3 $$\begin{aligned} \pi :M\rightarrow N. \end{aligned}$$

Thus, mathematically, we begin by casting a model of thermodynamics as a fibre bundle with one-dimensional fibres.^12^

Jauch’s definitions of work, energy, and heat can now be given more general geometric definitions:

- *Work is any one-form on M that annihilates vertical vectors, *$$\pi _*(X)=0$$$$\Rightarrow \omega (X)=0$$. This reflects the fact that work incorporates only observable degrees of freedom. It also implies the standard coordinate expression of work: in local adapted coordinates $$(U,V_1,\dots ,V_{n-1})$$ for *M*, it follows^13^ that $$\omega = -\sum _{i=1}^{n-1}P_idV_i$$ where each $$P_1,\dots ,P_{n-1}$$ is a smooth function on *M*.  
- *Energy is a local vertical coordinate function *$U:M\rightarrow \mathbb {R}$* in that if *$\pi _*(X)=0$* and **X≠ 0**, then **dU(X)≠ 0**.* In adapted local coordinates $(U,V_1,\dots ,V_{n-1})$ for *M* this is just what guarantees that energy is a coordinate variable. It says that it is always possible to have changes in energy without any changes in work.
- *Heat is locally defined by *$\xi := dU + \omega$*.* This means that heat is unobservable energy, in the sense that when one subtracts the change in observable energy $\sum _{i=1}^{n-1}P_idV_i$ from the change in total energy *dU*, the result is heat, $dU - \sum _{i=1}^{n-1}P_idV_i = dU + \omega = \xi$.

With these definitions, we will say as before that a curve *γ * is an *adiabat* if $\xi (\bar{\gamma })=0$, and is a *work cycle* if $\pi [\gamma ]$ is closed in *N*. The latter is a more general, coordinate-free way to express the statement that the curve *γ * starts and ends at the same point in the work configuration variables.

### Reformulation of the Conservation Law

The fibre bundle framework allows Jauch’s conservation law to be given a more informative expression. I will develop this in two steps. The first is to formulate it as a relationship between two kinds of closed loops. The second is to state it in the language of holonomies and curvature.

Let $\pi :M\rightarrow N$ be a fibre bundle with one-dimensional fibres, with work, energy, and heat defined as above. If *γ * is an adiabat, then $\xi (\bar{\gamma })=0$, which means that

4 $$\begin{aligned} \int _\gamma \omega = \int _\gamma (\xi - dU) = -\int _\gamma dU. \end{aligned}$$

So, whenever $\int _\gamma \omega =0$, it also follows that $U(\gamma (0))=U(\gamma (1))$. When it is also known that *γ * is a work cycle, this is exactly what is needed to conclude that *γ * is closed. Now, recall Jauch’s conservation law $(C_1)$: in a neighbourhood of each point, every adiabatic work cycle *γ * satisfies $\int _\gamma \omega =0$. We have just shown that this statement is equivalent to the following:

$(C_2)$: There is a neighbourhood of every point in which, if *γ * is an adiabat in *M* and $\pi [\gamma ]$ is closed in *N*, then *γ * is also closed.

**Fig. 3:**
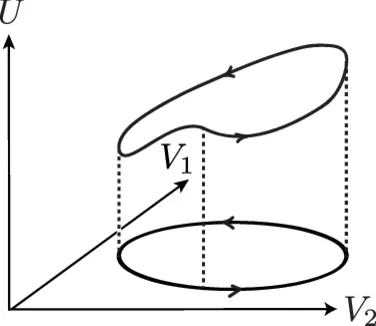
A geometric interpretation of Jauch’s conservation law: if an adiabat is also a work cycle, meaning it is closed in the $V_1-V_2$ plane, the curve itself must be closed

The loop-based expression of $(C_2)$ is highly suggestive of the holonomy formulation of Yang-Mills gauge theory.^14^ Readers familiar with that theory may indeed notice that our spiral in Fig. 2 looks suspiciously like a holonomy. Given a fibre bundle with an affine connection, a holonomy measures the extent to which a closed loop in the base space lifts to an open curve in the total space, like the spiral in Fig. 2. The failure to lift to a closed curve indicates the presence of curvature, as when one parallel transports a vector around a closed loop on the surface of a sphere: due to the curvature of the sphere, the closed loop in the base space lifts to an open curve in the tangent bundle. In this language, $(C_2)$ says that there are no non-trivial holonomies in the sense that every lifted loop closes, as depicted in Fig. 3.

To apply this thinking to thermodynamics, we would need a precise notion of a ‘lift’. In Yang-Mills theory it is defined using an (often affine) connection. However, a lift can also be defined using a more general Ehresmann connection, which is available in thermodynamics. Following [15], an *Ehresmann connection* is any sub-bundle *H⊆ TM* that decomposes the tangent bundle into horizontal and vertical components at every point,

5 $$\begin{aligned} T_pM = H_p \oplus V_p. \end{aligned}$$

The ‘vertical’ subspace $V_p$ here consists of those vectors *X* such that $\pi _*(X)=0$. The *curvature *$\Omega :H\times H\rightarrow V$ of an Ehresmann connection is the map that takes each *X,Y∈ H* to the vertical projection of the Lie bracket $\Omega (X,Y):={{\,\textrm{vert}\,}}[X,Y]$. Thus, as remarked above, curvature measures the extent to which horizontal parallel transport along infinitesimal loops in the base space fails to close in the bundle.

In any neighbourhood where the heat one-form *ξ * is non-vanishing, it defines an Ehresmann connection given by the ‘adiabatic’ distribution of vectors *X* satisfying *ξ (X)=0*, which we denote,

6 $$\begin{aligned} H=\ker \xi . \end{aligned}$$

This *H* is an Ehresmann connection essentially because vertical vectors are never adiabatic. If they were, then a non-zero vertical vector would satisfy both *ω (X)=0*, since we have assumed that *ω * annihilates vertical vectors, and *ξ (X)=0*. But then since $\xi =dU+\omega$, this would imply that *dU(X)=0*, which is impossible because *U* is a vertical fibre coordinate. So, a vertical process is never adiabatic, and $H_p\cap V_p=\{0\}$. But $\dim H_p=n-1$ (as is the case for the kernel of every non-vanishing one-form) and $\dim V_p=1$, and so $T_pM = H_p \oplus V_p$ as claimed. When $H=\ker \xi$ is the Ehresmann connection defining a set of adiabats in this way, I will call it an *adiabatic connection*.

For our purposes, the key feature of an Ehresmann connection is that it defines a lift from the base space *N* up to the bundle *M*. This draws on a standard theorem (cf. [15], Theorem 2.2, pp.69–70): for every curve $\gamma _N:I\rightarrow N$ and every point *p* in the fibre above $\gamma _N(0)$, there is a subinterval *J⊆ I* and a curve $\gamma :J\rightarrow M$ (called the *local horizontal lift* of $\gamma _N$ at *p*) such that (a) *γ * is horizontal, (b) $\pi (\gamma (t)) = \gamma _N(t)$ for all *t∈ J*, and (c) *γ (0)=p*; this *γ * is moreover uniquely determined by the three conditions (a)-(c). A horizontal lift is ‘local’ in the sense that it is only guaranteed to exist for some segment of the original curve given by *J⊆ I*. However, there is a class of Ehresmann connections that determine a ‘global’ lift for all parameter values, *J=I*, which are called *horizontally complete* connections. For example, if the total space *M* is compact, then a horizontally lifted curve is guaranteed to be a complete vector field, and so every Ehresmann connection is horizontally complete. It is common to assume horizontal completeness, and we will do so here. If $H=\ker \xi$ is horizontally complete, then *ξ * is automatically everywhere non-vanishing, and so no additional qualification of this kind is needed. We will thus drop the ‘local’ and simply say *horizontal lift*. In this paper we refer to the lift defined by an adiabatic connection as the *adiabatic lift*.

In summary: *a thermodynamic system defines a fibre bundle *$\pi :M\rightarrow N$*with one-dimensional fibres, together with a one-form **ξ ** for which *$H=\ker \xi$* is a horizontally complete Ehresmann connection. It defines adiabatic vectors (**X∈ H**) as well as adiabats (horizontal curves), and uniquely defines the horizontal (adiabatic) lift from a curve beginning at some point in the base space to each point in the fibre above it.* In this language, Jauch’s conservation law can be expressed as follows.

$(C_3)$: There is a neighbourhood *B⊆ M* of every point in which, if $\gamma _N$ is a closed curve in *π (B)* and its adiabatic lift *γ * begins and remains in *B*, then *γ * is closed in *B*.

Our reformulation $(C_3)$ says that the adiabatic connection only has locally trivial holonomies. As we will discuss in the next subsection, a connection with locally trivial holonomy can be shown to have zero curvature, which is commonly referred to as a *flat connection*. Thus, Jauch’s conservation hypothesis in this language is the assumption that the adiabatic connection is flat.

The discussion above suggests thermodynamics admits a formulation as a full-blown gauge theory. That is indeed the case, in the sense that the mathematical formalism of Yang-Mills theory for thermodynamics can be developed in much more detail as is done by [30]. However, our more modest aim in what follows is just to correct and complete the proof of Jauch’s theorem using some of these tools.

### Proof of the Theorem

Given a one-form $\xi = dU + \omega$ on a manifold *M*, Jauch’s theorem states that his conservation law implies the existence of local functions *T*, *S* such that *ξ = TdS*. We have now seen that these conditions generalise to a fibre bundle equipped with an adiabatic (Ehresmann) connection, where his conservation law is equivalent to $(C_3)$, which expresses that the adiabatic connection has locally trivial holonomies. This allows for a reformulation of Jauch’s theorem as a consequence of the Frobenius theorem, from which Jauch’s original theorem follows as a corollary:

#### Theorem 1

Let $\pi :M\rightarrow N$ be a fibre bundle with one-dimensional fibres, and let $H=\ker \xi$ be a horizontally complete Ehresmann connection. If, for some neighbourhood *B⊆ M* of every point, the adiabatic lift of every closed curve in *π (B)* that begins and remains in *B* is also closed, then in that neighbourhood there exist smooth functions *T*, *S* such that *ξ = TdS*.

#### Proof

We first show that our assumptions imply the curvature of *H* vanishes. Let *p∈ M* and let $X,Y\in H_p$. Let $U_N,V_N$ be local vector fields on *N* extending $\pi _*X$ and $\pi _*Y$, respectively, and which are constant in a local coordinate chart. Let *U*, *V* be their horizontal lifts. Then $[U_N,V_N]_{\pi (p)}=0$, which means that $[U,V]_p$ is vertical. Thus,

7 $$\begin{aligned} \Omega (X,Y) = {{\,\textrm{vert}\,}}[U,V]_p = [U,V]_p. \end{aligned}$$

Now, horizontal completeness guarantees that the horizontal lifts of sufficiently small rectangles generated by $U_N, V_N$ exist. By choosing these rectangles to be sufficiently small so that their lifts remain in *B*, it follows from our hypothesis that the lifts of all such rectangles close. Thus, the corresponding local flows generated by the lifted vector fields *U* and *V* commute, and so by a standard result their commutator vanishes, $[U,V]_p=0$. Therefore, $\Omega (X,Y)=[U,V]_p = 0$.

An Ehresmann connection with zero curvature is always involutive [15, Theorem 2.1, p.87]. Moreover, by the Frobenius theorem [6, Theorem 1.1], $H=\ker \xi$ is involutive if and only if it is integrable. So, by choosing *S* to be a function such that *dS=0* on the integral submanifolds of *H*, it follows that since the one-forms *dS* and *ξ * have the same kernel, they differ only by a scalar field *T*, from which we conclude that *ξ = TdS*. *□ *

Jauch’s original theorem can now be derived by retracing the discussion above: given local coordinates $(U,V_1,\dots ,V_{n-1})$ for *M* and one-forms $\omega = -\sum _{i=1}^{n-1}P_idV_i$ and $\xi = dU + \omega$, we have seen that the projection onto the coordinates $(V_1,\dots ,V_{n-1})$ defines a fibre bundle $\pi :M\rightarrow N$. Moreover, the subbundle $H = \ker \xi$ is an Ehresmann connection when *ξ * is non-vanishing. Jauch’s conservation law $(C_1)$ now says that an adiabatic work cycle produces no work. This implies $(C_2)$, that if an adiabat *γ * projects onto a closed loop $\pi [\gamma ]$ in *N* then *γ * is closed in *M*. And, that implies $(C_3)$, that every adiabatic lift of a closed curve in *N* is closed in *M*. By Theorem 1, it follows that *ξ =TdS* for some local functions *T* and *S*, and so Jauch’s theorem is proved.

This discussion shows that, like Carathéodory’s theorem,^15^ Jauch’s theorem is fundamentally local. Indeed, the conclusion need not hold globally on non-contractible manifolds, analogous to the circumstances that give rise to a geometric phase in Yang-Mills gauge theories. The possibility of geometric phase is not by itself a failure of the framework, and indeed in electromagnetism it was associated with the new physical prediction of the Aharonov-Bohm effect; for a discussion of the empirical significance of geometric phase in thermodynamics, see [30].

What are we to say about those systems in which the premises of this theorem do not apply? As we saw in Section 2, Jauch’s conservation law applies to those systems that are ‘conservative’, in the sense that restoring the initial work configuration variables along an adiabat does not result in a change in energy. This statement now takes an elegant mathematical form: it says that the adiabatic connection has zero curvature. It also immediately suggests a possible way to model irreversible systems in which heat cannot be written in terms of entropy and temperature: they are described by adiabatic connections with non-zero curvature. This would seem to be a remarkable pathway for future study opened by the bundle framework for thermodynamics.

## Connection to Tatiana Afanassjewa

Before concluding, let me briefly discuss the relationship between Jauch’s foundation for thermodynamics and the one proposed by Tatiana Afanassjewa [11, p.223]. In an early remark about his result, Jauch said that it was “discovered by T. Ehrenfest” [19], referring to a pair of papers she published in 1925 and 1926. I have not been able to find any evidence of Jauch’s theorem in these papers, or elsewhere in Afanassjewa’s work. Instead, she consistently relies on Carathéodory’s theorem, which is a different statement that is proved in a different way.

However, Jauch does appear to have been inspired by her thesis that “the irreversibility of non-static processes has no relevance for the existence of entropy” [11, p.178], which she defended throughout her career. Jauch also thought that the lack of a physical basis for her axioms kept this observation from being widely accepted.^16^ I would like to point out that Jauch’s conservation hypothesis in fact follows from Afanassjewa’s ‘fundamental hypothesis’, which she calls *Axiom A* (the Entropy Axiom). She stated her axiom as follows:

“If the integral $\int _1^2 \delta Q$ is non-zero along a quasi-static path that connects states 1 and 2 of a thermally homogeneous system, then the system cannot be brought from one of these states into the other along an adiabatic quasi-static path.” [11, p.172]

As Jauch points out, the physical motivation for this principle could be further developed. But it serves her purposes because it both avoids the assumption of irreversibility and also implies Carathéodory’s principle.^17^ Thus she concludes that heat can be written *ξ =TdS* by Carathéodory’s theorem.

To compare Afanassjewa’s thinking more precisely to the picture we have developed here, suppose we can take her to interpret every point in thermodynamic state space *M* as an equilibrium state. Then, every curve in *M* describes a process that is *quasi-static*, meaning a sequence of equilibrium states.^18^ Then Afanassjewa’s axiom states, *if *$\int _\gamma \xi \ne 0$* along a (quasi-static) curve that connects p and q*, *then p and q cannot be connected by any (quasi-static) adiabat*, or in equivalent contrapositive form,

(A) If *p* and *q* are adiabatically connected by a (quasi-static) curve, then $\int _\gamma \xi = 0$ on every (quasi-static) curve *γ * from *p* to *q*.

**Fig. 4:**
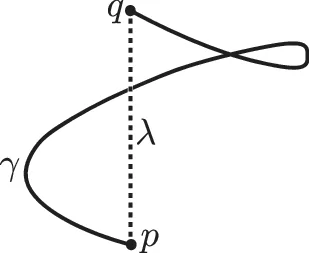
An adiabatic work cycle *γ * defining a vertical curve *λ *

In contrast, the derivation of entropy and temperature from Jauch’s conservation law does not require us to introduce a notion of equilibrium states or quasi-static processes. I see this high degree of generality as a virtue of the account.^19^ However, it is compatible with these concepts. In fact, Afanassjewa’s axiom (A) implies a special case of Jauch’s conservation law $(C_2)$, where the special case is one in which every state is interpreted as equilibrium, and every curve is interpreted as quasi-static. For suppose there is a (quasi-static) adiabatic work cycle *γ * from *p* to *q*. Since a work cycle satisfies $\pi (p)=\pi (q)$, there must be a vertical (quasi-static) curve *λ * from *p* to *q*, as in Fig. 4, which by (A) satisfies $\int _\lambda \xi =0$. But, since *λ * is vertical, it must satisfy $\int _\lambda \omega =0$ as well, which means that $\int _\lambda dU=0$, and hence *U(p)=U(q)*. Thus, *p=q*, and the original (quasi-static) adiabatic work cycle *γ * must be closed.

Since the converse does not hold, Jauch’s conservation law is strictly weaker than Afanassjewa’s axiom, even in the special context in which every state is assumed to be equilibrium. It also means that our result is strictly stronger than hers, in that Theorem 1 reaches the same conclusion by assuming less.

That said, the fact that entropy can be derived without irreversibility leaves open a number of questions about irreversibility. These include the origin of irreversibility as it appears in the Minus-First law [5], as well as the apparent incompatibility between thermodynamic irreversibility and the reversibility of most models of classical statistical mechanics. An optimistic appraisal of these issues can be found in [29]. However, more work remains to be done regarding the exact relationship between thermodynamics and statistical mechanics from this perspective.

## Conclusion

Jauch’s conservation law does indeed provide a new foundation for thermodynamics, which has many virtues: its physical implications are clear and plausible, it avoids appeal to ‘adiabatic accessibility’, and it does not depend on any irreversible ingredient like the second law. The essential structures it requires are just the distinction between work and total (internal) energy, and the undirected notion of an adiabat. In this sense, Jauch’s approach appears to have advantages over many other approaches to deriving entropy and temperature.

Although Jauch’s own line of argument unfortunately did not succeed in establishing this, a new and more revealing expression of his conservation law does enable a successful proof. Most remarkably, this new proof involves viewing thermodynamics as a gauge theory, and in particular as a fibre bundle with an Ehresmann connection interpreted as defining the adiabats. In that framework, Jauch’s conservation law becomes the statement that there are only trivial holonomies, which is to say that the adiabatic connection is flat. This immediately implies the integrability condition needed to ensure that heat can be written as *TdS*. Our framework thus converts Jauch’s theorem into an elegant part of the geometric structure of thermodynamics. That geometric structure itself appears to be an interesting new area of potential research.

## Data Availability

No datasets were generated or analysed during the current study.

## Footnotes

1 This is especially true for entropy, but also for temperature: one should not be too cavalier about defining temperature as ‘just what is measured by thermometers’ before reviewing the extraordinary conceptual challenge of defining thermometers, as breathtakingly chronicled by Chang [10].

2 Common wisdom that temporal direction arises from entropy increase or the second law has been challenged by [5, 14, 23, 27, 33], and [28, Chapter 6].

3 Jauch appears to have stumbled onto this idea in a coauthored paper on Szilard’s Paradox where they write in a footnote: “It seems not to be generally known that the existence of the integrating factor, hence the existence of the function entropy, is a consequence of the first principle of thermodynamics for conservative systems under reversible quasistatic variations. This was discovered by T. Ehrenfest. A new proof of this statement will be given in a subsequent publication” [19, p.223].

4 For example, see [12, 18, 20, 28, 33, 34, 37].

5 Some of the formal structures that I draw on for this proof are developed in more detail in [30], where it is argued that thermodynamics can in general be viewed as a gauge theory. In contrast, the purpose of the present paper is just to develop and correct Jauch’s foundation for the origin of entropy.

6 Carathéodory wrote that “in order to be able from the outset to treat systems with arbitrarily many degrees of freedom, instead of the Carnot cycle that is almost always used, but is intuitive and easy to control only for systems with two degrees of freedom, one must make use of a theorem from the theory of Pfaffian differential equations” [8, p.357]. For more concerns about the Carnot cycle approach see [33, §6.2].

7 Throughout this paper we will make no mathematical assumption to the effect that each state is one of ‘equilibrium’ or that each curve must be ‘quasi-static’. These concepts are compatible with the present discussion but will not be necessary. We introduce these concepts only superficially in Section 5 in order to compare Jauch’s conservation law to Afanassjewa’s Axiom (A).

8 One explicit such statement by Carnot is: “The result of these first operations has been the production of a certain quantity of motive power and the removal of caloric from the body *A* to the body *B*. The result of the inverse operations is the consumption of the motive power produced and the return of the caloric from the body *B* to the body *A*; so that these two series of operations annul each other, after a fashion, one neutralizing the other” [9, pp.66-67].

9 I adopt the sign convention that *ω =-PdV* represents ‘inward-directed’ work done on the system, while *-ω =PdV* represents the work performed by the system on its environment.

10 *Proof:* Consider a curve *γ * with tangent vector field $\bar{\gamma }$ that is an adiabat, $0 = \xi (\bar{\gamma })=(dU - PdV)(\bar{\gamma })$. When this curve is written in local coordinates as *U*(*V*), it is a solution to the ODE, *P = dU/dV*. So, the local existence and uniqueness theorems imply that each point admits a neighbourhood that is foliated by such solutions, each of which is an adiabat. Now, let *S* be any function that is constant along each leaf of the foliation (that is, on each adiabat). Then for every vector *X* in the neighbourhood, *dS(X)=0* if and only if *ξ (X)=0*. But, a non-vanishing one-form is determined up to a scale factor by its kernel. Thus, in that neighbourhood, there is a smooth function *T* such that *ξ = TdS*. In contrast, with more volume terms like $\xi =dU - P_1dV_1 - P_2dV_2$, adiabats are characterised by a Pfaffian equation for which no comparable result is available.

11 See [8], as well as [3, 4], or [6, Theorem 3.5] for a rigorous proof.

12 We assume by definition that a fibre bundle admits a local trivialisation. However, we do not necessarily assume that the bundle is globally trivial; in particular, we assume only the possibility of a local (but not necessarily global) energy function *U*.

13 A one-form that annihilates all vertical vectors is called *semi-basic* and has the property that $\omega = -\sum _{i=1}^{n-1}P_i dV_i$ [24, Def. 7.4.2].

14 The philosophical significance of the holonomy formulation has been the subject of much discussion; see [2, 13, 25, 32], and [16].

15 The local nature of Carathéodory’s theorem was pointed out by [3] and its implications discussed by [4] and [21, §10.3.2].

16 Jauch wrote, “she pointed out that the existence of an integrating factor for the differential 1-form representing the reversible heat transmitted to a thermal system has nothing to do with the irreversibility of all spontaneous processes occurring in time. ... A conceivable reason for the lack of interest in T. Ehrenfest’s important remark may be that her fundamental hypothesis, which replaces the second principle of thermodynamics, has no concrete physical interpretation” [17, p.328].

17 For evidence that Carathéodory’s principle already avoids any irreversible ingredient see [23].

18 This interpretation is indeed strictly needed for Afanassjewa to avail herself of Carathéodory’s theorem in the way she does, since the precise expression of that theorem is formulated on a smooth manifold *M* with a one-form *ξ * representing heat, and where an accessibility relation is defined in terms of arbitrary curves in *M*.

19 Indeed, the precise meaning of quasi-static processes is a non-trivial interpretive issue, as Norton [26] has pointed out. For an alternative appraisal see [36] and [31].

## References

[1] Albert, D.Z.: Time and chance. Harvard University Press, Cambridge, MA (2000)

[2] Belot, G.: Understanding electromagnetism. Br. J. Philos. Sci. **49**(4), 531–555 (1998)

[3] Bernstein, B.: Proof of Carathéodory’s local theorem and its global application to thermostatics. J. Math. Phys. **1**(3), 222–224 (1960)

[4] Boyling, J.: Carathéodory’s principle and the existence of global integrating factors. Commun. Math. Phys. **10**(1), 52–68 (1968)

[5] Brown, H. R., Uffink, J.: The Origins of Time-Asymmetry in Thermodynamics: The Minus First Law. Studies in History and Philosophy of Modern Physics **32**(4), 525–538 (2001). http://philsci-archive.pitt.edu/217/

[6] Bryant, R.L., Chern, S., Gardner, R., Goldschmidt, H., Griffiths, P.: Exterior Differential Systems. Springer-Verlag, New York Inc, New York (1991)

[7] Buchdahl, H.A.: The Concepts of Classical Thermodynamics. Cambridge University Press, Cambridge (1966)

[8] Carathéodory, C.: Untersuchungen über die Grundlagen der Thermodynamik. Math. Ann. **67**, 355–386 (1909)

[9] Carnot, S.: Reflections on the Motive Power of Heat, London: Macmillan & Co. 1890 English Translation by R.H. Thurston (1824). https://www.google.co.uk/books/edition/Reflections_on_the_motive_power_of_heat/_tHxDntE2GQC

[10] Chang, H.: Inventing Temperature: Measurement and Scientific Progress. Oxford University Press, New York (2004)

[11] Ehrenfest-Afanassjewa, T.: Zur Axiomatisierung des zweiten Hauptsatzes der Thermodynamik, Zeitschrift für Physik **33**, 933–945. Translated into English by Marina Baldissera Pacchetti as, ‘On the Axiomatization of the Second Law of Thermodynamics’, in Uffink et al. (2021) The Legacy of Tatiana Afanassjewa, Springer Nature Switzerland AG, pp.170–179 (1925)

[12] Emch, G.G.: Mathematical and conceptual foundations of physics. Elsevier Science Publishers B.V, Amsterdam (1984)

[13] Healey, R.: Gauging What’s Real: The Conceptual Foundations of Contemporary Gauge Theories. Oxford University Press, New York (2007)

[14] Henderson, L.: Can the second law be compatible with time reversal invariant dynamics? Studies in History and Philosophy of Modern Physics **47**, 90–98 (2014). http://philsci-archive.pitt.edu/10698/

[15] Hermann, R.: Gauge fields and Cartan-Ehresmann Connections. Math Sci Press, Part A, Brookline, MA (1975)

[16] Jacobs, C.: The metaphysics of fibre bundles. Studies in History and Philosophy of Science **97**, 34–43 (2023). http://philsci-archive.pitt.edu/21468/10.1016/j.shpsa.2022.11.010 36525712

[17] Jauch, J.M.: On a new foundation of equilibrium thermodynamics. Found. Phys. **2**(4), 327–332 (1972)

[18] Jauch, J.M.: Analytical Thermodynamics, Part 1: Thermostatics—General Theory. Found. Phys. **5**(1), 111–132 (1975)

[19] Jauch, J.M., Báron, J.: Entropy, information and Szilard’s paradox. Helv. Phys. Acta **45**(2), 220–232 (1972)

[20] Kuzemsky, A.: In search of time lost: Asymmetry of time and irreversibility in natural processes. Found. Sci. **25**(3), 597–645 (2020)

[21] Lavis, D.A., Frigg, R.: The fundamentals of thermodynamics. Springer Nature Switzerland AG, Cham, Switzerland (2025)

[22] Lieb, E. H., Yngvason, J.: The physics and mathematics of the second law of thermodynamics. Physics Reports **310**(1), 1–96 (1999). arxiv.org/abs/cond-mat/9708200

[23] Marsland III, R., Brown, H.R., Valente, G.: Time and irreversibility in axiomatic thermodynamics. Am. J. Phys. **83**(7), 628–634 (2015)

[24] Montgomery, R.: A Tour of Subriemannian Geometries. Their Geodesics and Applications, The American Mathematical Society, Providence, RI (2002)

[25] Myrvold, W. C.: Nonseparability, classical, and quantum. The British Journal for the Philosophy of Science **62**, 417–432 (2011). https://philsci-archive.pitt.edu/5335/

[26] Norton, J. D.: The impossible process: Thermodynamic reversibility. Studies in History and Philosophy of Modern Physics **55**, 43–61 (2016). http://philsci-archive.pitt.edu/12341/

[27] Price, H.: Time’s Arrow and Archimedes’ Point: New Directions for the Physics of Time. Oxford University Press, New York (1996)

[28] Roberts, B. W.: Reversing the Arrow of Time. Cambridge: Cambridge University Press (2022). Open Access: 10.1017/9781009122139

[29] Roberts, B. W.: Does thermodynamics have a reversibility problem? Mind **Forthcoming**, 1–14 (2026a). Open Access: 10.1093/mind/fzag008

[30] Roberts, B. W.: Heat as a gauge connection. Unpublished Manuscript (2026b): arxiv.org/abs/2503.08753

[31] Robertson, K.: In Search of the Holy Grail: How to Reduce the Second Law of Thermodynamics. The British Journal for the Philosophy of Science **73**(4), 987–1020 (2022). https://philsci-archive.pitt.edu/17516/

[32] Rosenstock, S., Weatherall, J. O.: A categorical equivalence between generalized holonomy maps on a connected manifold and principal connections on bundles over that manifold. Journal of Mathematical Physics **57**(10), 102902 (2016). http://philsci-archive.pitt.edu/11904/

[33] Uffink, J.: Bluff your way in the second law of thermodynamics. Studies In History and Philosophy of Modern Physics **32**(3), 305–394 (2001). http://philsci-archive.pitt.edu/313/

[34] Uffink, J.: Compendium of the Foundations of Classical Statistical Physics. In: Butterfield, J., Earman, J. (eds.), Philosophy of Physics Part B, Elsevier, pp. 923–1074 (2007). http://philsci-archive.pitt.edu/2691/

[35] Uffink, J., Valente, G.: Afanassjewa and the foundations of thermodynamics. In: Uffink, J., Valente, G., Werndl, C., Zuchowski, L. (eds.), The Legacy of Tatjana Afanassjewa, Cham, Switzerland: Springer Nature Switzerland AG, chapter 3, pp. 55–82 (2021)

[36] Valente, G.: On the paradox of reversible processes in thermodynamics. Synthese **196**(5), 1761–1781 (2019)

[37] Walter, J.: On the definition of the absolute temperature—a reconciliation of the classical method with that of Carathéodory. Proc. R. Soc. Edinb. **82A**, 87–94 (1978)

